# Distinguishing the taphonomic signature of wolves from humans and other predators on small prey assemblages

**DOI:** 10.1038/s41598-020-64716-8

**Published:** 2020-05-15

**Authors:** Lluís Lloveras, Jordi Nadal, Josep Maria Fullola

**Affiliations:** SERP. Departament d’Història i Arqueologia. Universitat de Barcelona. C/Montalegre 6-8, E-08001 Barcelona, Spain

**Keywords:** Environmental social sciences, Palaeontology

## Abstract

The study of human subsistence strategies in prehistoric hunter-gatherer communities is essential to understanding the evolution of human behaviour. An important topic of interest is the expansion of dietary breadth, resulting in the procurement of a larger number of small game species. However, to make accurate interpretations of human subsistence, the correct identification of the agents responsible for archaeofaunal assemblages is crucial, and actualistic studies that establish the taphonomic signature of the different predators are indispensable. Despite being one of the most ubiquitous carnivores in prehistoric archaeological sites, the role of wolves (*Canis lupus*) as agents responsible for small-prey accumulations has never been examined. The aims of this study are to analyse the taphonomic patterns left by wolves on rabbit remains and to put forward a series of criteria that can help distinguish assemblages produced by this carnivore from those accumulated by people or by other predators. Our results reveal that wolves ingest and consume the whole rabbit carcass, with the consequence that all rabbit remains accumulated by wolves come from the scats. The referential framework provided in this study will make it possible to discriminate wolves as agents of fossil rabbit accumulations.

## Introduction

The study of human subsistence strategies in prehistoric hunter-gatherer communities is essential to understanding the evolution of human behaviour. Of particular note among the topics related to human subsistence still under debate is the *broad spectrum revolution* theory^1^, which proposes that an expansion of dietary breadth led to the procurement of a larger number of species, particularly through the greater exploitation of small game^2–5^. The substantial introduction of small animals into the human diet in specific periods and/or regions is considered to have been an important advantage in the subsistence of hunter-gatherer groups, facilitating population growth and territorial expansion^3,4,6^.

The development of dietary breadth was first detected in southern Europe and the Levant during the early Upper Palaeolithic and was related to the expansion of anatomically modern humans. However, more recent studies have also shown evidence of different types of small-prey use prior to the arrival of anatomically modern humans in Europe^7–12^. These studies suggest that the evolution of dietary breadth was not linear and that foraging strategies were more diverse than previously thought, varying according to a combination of different factors such as climate conditions and prey availability, demographic pressure, technological advantages or energy return rates^13,14^.

To identify the agents responsible for archaeofaunal assemblages correctly, it is crucial to make accurate interpretations of past human subsistence. This is especially relevant in studies dealing with small-prey remains, given that there were a large number of carnivores that were active accumulators of animal bones in caves and shelters shared with humans. A good example is the case of the rabbit (*Oryctolagus cuniculus*), the most important small prey in many areas such as the Iberian Peninsula or the South of France, and an important source of food for a large number of predator species^15^. In recent decades, following the analytical methodology developed by Andrews^16^, many systematic actualistic studies of modern small-prey assemblages accumulated by different predators have been conducted, examining the role of carnivores as possible agents of bone accumulation in archaeological deposits. Rabbits, because of their importance, have been the focus of many of these studies^17–27^. However, despite being one of the most ubiquitous carnivores in prehistoric archaeological sites, the role of the wolf (*Canis lupus*) as an agent responsible for accumulations of small-prey remains has never been examined. Wolves are a widespread Holarctic species distributed across a broad variety of habitats, including deserts, dry plains, boreal forests, and the High Arctic^28^. They are generalist carnivores, feeding on a wide variety of species throughout their range. Wild ungulates (e.g. red deer, roe deer, fallow deer, moose, wild boar) tend to be their preferred prey, but normally they also prey on smaller animals such as small carnivores, hares, beavers, squirrels, rabbits or birds^29–31^. Rabbits are a recurrent prey, reaching values of up to 45% of the wolf diet in some areas where these animals are abundant^31–34^. Besides, wolves can use caves and rock shelters, particularly during the breeding season, where they deposit their food debris^35^. Studies about wolves’ behaviour demonstrate that, despite not accumulating bones in the same large quantities as other carnivores do^36^, parts of their prey carcass (including bones, antlers, and hair) are often brought by the animals to the den from a kill site^37^. These studies also show that abundant scats containing prey digested teeth and bone fragments can be found around and inside the caves used as dens^38,39^. This indicates that wolves might well have been agents responsible for rabbit bone assemblages at archaeological sites.

The aims of this study are as follows: firstly, to study the taphonomic patterns left by the wolf on rabbit remains; and secondly, to put forward a series of criteria that can help distinguish assemblages produced by wolves from those accumulated by people or by other predators in archaeological samples.

To this end, an experimental study was conducted with an adult male Iberian wolf (*Canis lupus signatus*) kept at the wildlife recovery centre, the Centro de Naturaleza Cañada Real (Peralejo, Spain). During March and April 2013, the wolf was fed with 15 complete domestic rabbit carcasses. The rabbit remains used in this study were from a farm specialized in breeding rabbits. All the animals used in the study were sub-adults. The protocol integrated the recovery of the rabbit leftovers not ingested during the feeding as well as the scats deposited inside the enclosure for subsequent analysis. Before each feeding episode the predator enclosure was cleaned of previous meals and scats.

## Results

**Figure 1:**
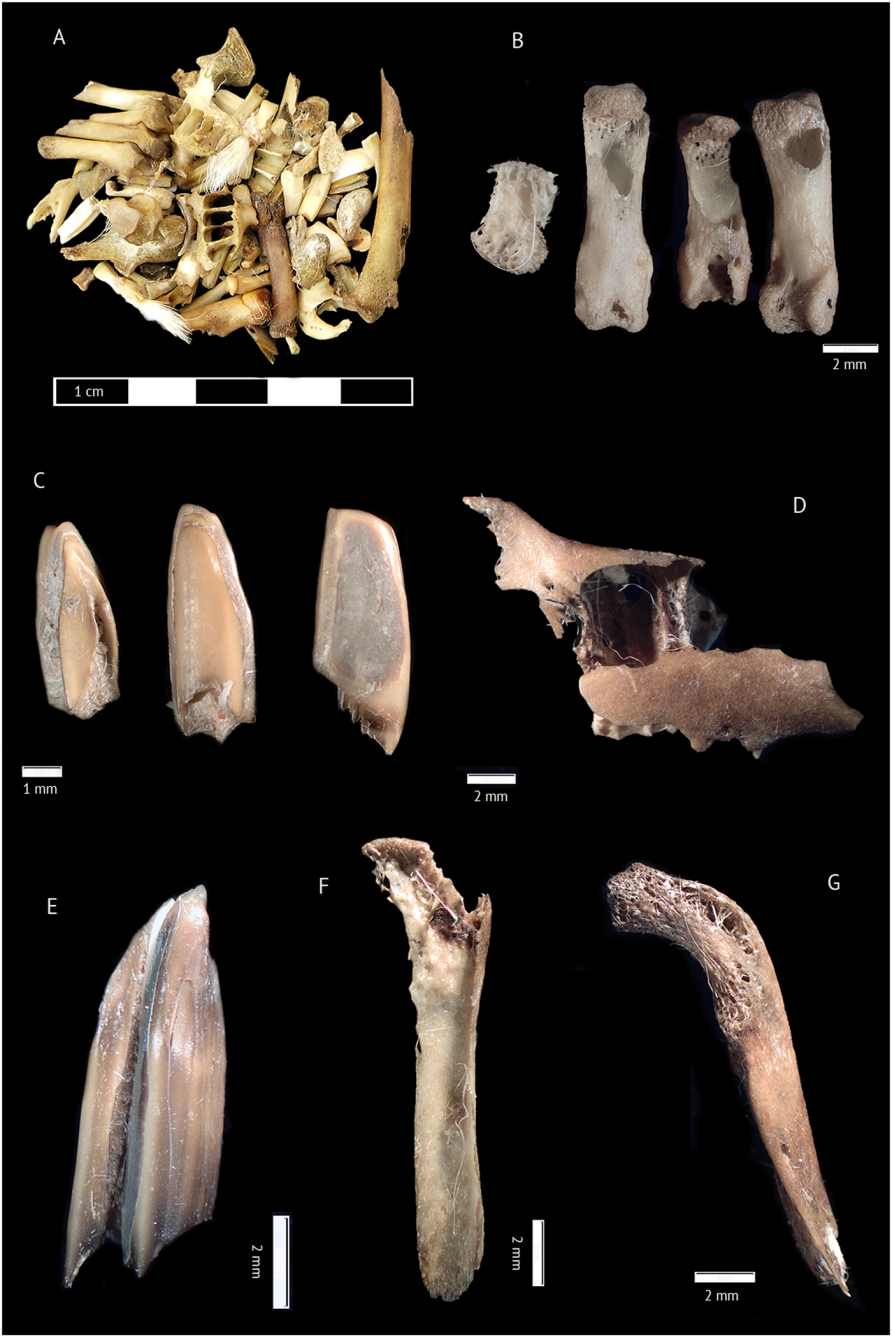
Example of highly fragmented and digested rabbit bones and teeth recovered from a wolf scat (**A**). First and second phalanges affected by extensive digestive corrosion damage (**B**). Rabbit molars showing extreme, heavy and moderate degree of digestive damage (**C**). Fragment of mandible (**D**). Lower molar with extreme digestive damage (**E**). Metapodial (**F**). Ulna (**G**).

The wolf ingested and consumed the whole rabbit carcass, with the result that rabbit leftovers not ingested during the feeding were not found. Therefore, all the rabbit remains analysed here were recovered from the wolf scats (Fig. 1).

### Anatomical representation

A total of 935 bones and teeth could be identified to anatomical part (Table 1). The estimated minimum number of individuals (MNI) was twelve. This MNI indicates an important bias of the expected value, which would be fifteen, the number of rabbits used in the study. The loss of more than 20% of the record is indicative of the significant destruction of the rabbit carcasses caused by the wolf during consumption.

**Table 1:** The number of identified specimens (NISP), number of identified specimens percentage (NISP%), minimum number of elements (MNE), minimum number of individuals (MNI), and relative abundance proportions (RA%) of rabbit remains recovered from the wolf scat sample. Digestive damage: numbers (N) and percentage (%) of rabbit bones included in each digestion category.

Wolf scats (MNI = 12)	Anatomical representation				Digestion damage									
					Null		Light		Moderate		Heavy		Extreme	
	NISP	NISP%	MNE	RA%	N	%	N	%	N	%	N	%	N	%
Mandible	28	3.0	9	37.5	0	0	0	0	1	3.6	6	21.4	21	75.0
Cranium	96	10.3	9	75	1	1.0	1	1.0	8	8.3	42	43.8	44	45.8
Incisors	32	3.4	32	64.6	0	0	0	0	9	28.1	18	56.3	—	15.6
Upper molar	139	14.9	139	96.5	0	0	0	0	44	31.7	54	38.8	—	29.5
Lower molar	83	8.9	83	92.3	0	0	0	0	12	14.5	54	65	—	20.5
Humerus	12	1.3	6	25	0	0	0	0	0	0	3	25	9	75
Radius	5	0.5	2	8.3	0	0	0	0	0	0	3	60	2	40
Ulna	1	0.1	1	4.2	0	0	0	0	0	0	0	0	1	100
Femur	10	1.1	4	16.7	0	0	0	0	0	0	1	10	9	90
Tibia	15	1.6	6	25	0	0	0	0	0	0	3	20	12	80
Patella	7	0.7	7	29.2	0	0	0	0	2	28.6	5	71.4	0	0
Scapula	2	0.2	2	8.3	0	0	0	0	0	0	0	0	2	100
Innominate	7	0.7	5	20.8	0	0	0	0	0	0	0	0	7	100
Metacarpus	3	0.3	3	2.5	0	0	0	0	1	33.3	1	33.3	1	33.3
Metatarsus	5	0.5	5	5.2	0	0	0	0	1	20	4	80	0	0
Metapodial	25	2.7	12	—	0	0	0	0	4	16	9	36	12	48
Phalanges 1/2	121	12.9	95	23.3	8	6.6	9	7.4	24	19.8	41	33.9	39	32.2
Phalanx 3	155	16.6	155	71.8	9	5.8	17	11.0	27	17.4	79	51.0	23	14.8
Calcaneum	7	0.7	4	16.7	0	0	0	0	0	0	1	14.3	6	85.7
Astragalus	2	0.2	2	8.3	0	0	0	0	0	0	0	0	2	100
Carpal/tarsal	8	0.9	7	2.4	0	0	0	0	1	12.5	1	12.5	6	75
Vertebra	155	16.6	85	15.4	0	0	2	1.3	22	14.2	55	35.5	76	49.0
Rib	17	1.8	10	3.5	0	0	0	0	1	5.9	6	35.2	10	58.8
TOTAL	935	—	683	—	18	—	29	—	157	—	386	—	345	—

**Figure 2:**
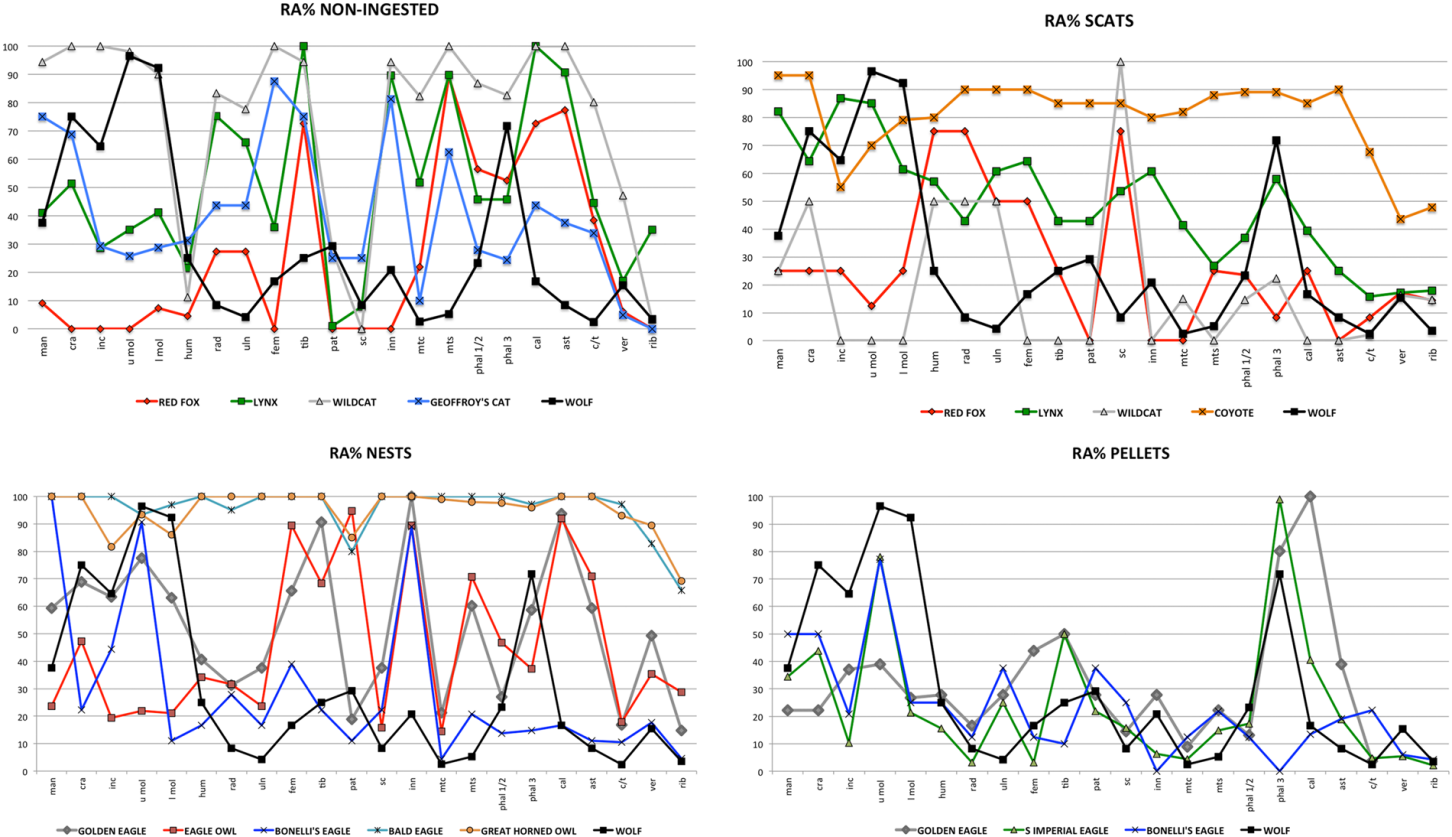
Comparison of relative abundance profiles of different predators (terrestrial carnivores and raptors) with wolf. Abbreviations: man mandible, cra cranium, inc incisors, u mol upper molars, l mol lower molars, hum humerus, rad radius, uln ulna, fem femur, tib tibia, pat patella, sc scapula, inn innominate, mtc metacarpals, mts metatarsals, phal 1/2 phalanges 1/2, phal 3 phalanx 3, cal calcaneum, ast astragalus, c/t carpal/tarsal, ver vertebrae, rib ribs.

The whole skeleton was represented but in very different proportions. In absolute numbers, phalanges (29.5%), vertebrae (16.6%) and upper molars (14.9%) were the most abundant elements (N%). The relative abundance of skeletal elements (RA%) is also shown in Table 1 and Fig. 2. The RA mean value (29.7%) was low, indicating a high loss of skeletal elements in the accumulation. Molars, the cranium, third phalanges and incisors, all of which displayed values over 70%, were the best-represented elements. Long bones were less represented, with an average RA value of 15.8%. Carpal/tarsal bones and ribs were the least represented (2.4% and 3.5% respectively).

The relative proportion indices of the skeletal elements indicate a deficiency in the numbers of postcranial compared to cranial remains (PCRT/CR = 22.7 ± 0.4; PCRAP/CR = 24.6 ± 0.4; PCRLB/CR = 18.3 ± 1.3). Among the limb bones, specimens from the lower appendicular skeleton were better represented than the upper ones (AUT/ZE = 57.1 ± 1.3). On the whole, zygopodia (radii/ulnae/tibiae) were less represented than stylopodia (humeri/femora) (Z/E = 37.5 ± 9.6). Posterior limb elements were better preserved than anterior elements (AN/PO = 37.5 ± 4.8).

### Breakage

The analysis of breakage revealed a high level of destruction in the sample, which mainly included very small fragments. Only a minority of specimens (15.3%) showed length values over 10 mm. The percentage of complete bones was 44.8%, but all complete elements consisted of small specimens such as molars or phalanges. Larger elements such as long bones, scapulae, pelvises, mandibles or crania were never recovered complete (Fig. 1).

The breakage categories showed the following features (Table 2):

- Long bone fragments were mostly represented by portions of the proximal epiphysis. Shaft cylinders were absent in the sample.
- Metapodials survived better, 15% being complete; most fragments comprised the distal epiphysis (42.4%).
- Most of the identified skull fragments were parts of the neurocranium (73.9%) and zygomatic arch (16.3%).
- In mandibles, body (64.3%) and fossa (32%) fragments were better represented than other fragments.
- In the innominates, most fragments contained the acetabulum (85.8%).
- Scapula fragments always comprised the fossa (NF and F).
- The frequency of complete vertebrae was 3.2%. Most fragments included the vertebral epiphyses and vertebral body.
- The ribs were never complete.
- Complete carpals/tarsals and third phalanges reached values of 87.1% and 87.5%. First and second phalanges were complete in 53.7% of cases. Patellae were always complete. Calcanea and astragali were always fragmented.
- Regarding teeth, molars were complete in percentages above 80% but incisors only in 40.6% of cases.

**Table 2:** Numbers and percentages of parts of skeletal elements included in each breakage category for the rabbit remains recovered from the wolf scat sample. Long bones, metacarpal and metatarsal bones were classified as complete (C), proximal epiphysis (PE), proximal epiphysis + shaft (PES), shaft (S), shaft + distal epiphysis (SDE) and distal epiphysis (DE). Mandibles as C, incisive part (IP), mandible body + incisive part (MBI), mandible body (MB), mandible body + branch (MBB) and condylar process (CP). Crania as C, incisive bone (IB), incisive bone + maxilla (IBM), maxilla (M), zygomatic arch (ZA) and neurocranium (NC). Innominates as C, acetabulum (A), acetabulum + ischium (AIS), acetabulum + ischium + ilium (AISIL), acetabulum + ilium (AIL), ischium (IS) and ilium (IL). Scapulae as C, glenoid cavity (GC), glenoid cavity + neck (GCN), glenoid cavity + neck + fossa (GCNF), neck + fossa (NF) and fossa (F). Vertebrae as C, vertebral body (VB), vertebral epiphysis (VE) and spinous process (SP). Phalanges as C, proximal fragment (P), distal fragment (D) and fragment (F). Patellae, carpals/tarsals, calcanea, astragali, ribs and teeth as C and F.

BREAKAGE CATEGORIES												
Long bones and metapodial	C		PE		PES		S		SDE		DE	
	N	%	N	%	N	%	N	%	N	%	N	%
Humerus	0	0	7	58.3	1	8.3	2	16.6	0	0	2	16.6
Radius	0	0	3	60	0	0	2	40	0	0	0	0
Ulna	0	0	0	0	1	100	0	0	0	0	0	0
Femur	0	0	6	60	0	0	2	20	0	0	2	20
Tibia	0	0	9	60	0	0	3	20	0	0	3	20
Metapodial	5	15.2	1	3	5	15.2	8	24.2	0	0	14	42.4
**Mandible**	**N**	**%**	**Cranium**	**N**	**%**	**Innominate**	**N**	**%**	**Scapula**	**N**	**%**	
C	0	0	C	0	0	C	0	0	C	0	0	
IP	1	3.6	IB	2	2.2	A	2	28.6	GC	0	0	
MBI	0	0	IBM	0	0	AIS	1	14.3	GCN	0	0	
MB	18	64.3	M	7	7.6	AISIL	3	42.9	NF	1	50	
MBB	9	32	ZA	15	16.3	AIL	0	0	F	1	50	
PC	0	0	NC	68	73.9	IS	0	0	IL	1	14.3	
**Vertebrae**	**N**	**%**	**Ribs**	**N**	**%**	**Phalanges 1/2**	**N**	**%**	**Phalanx 3**	**N**	**%**	
C	5	3.2	C	0	0	C	65	53.7		C	135	87.1
VB	38	24.5	F	17	100	P	26	21.5		F	20	12.9
VE	81	52.3				D	30	24.8				
SP	31	20.0										
**Patella**	**N**	**%**	**Car/tar**	**N**	**%**	**Cal**	**N**	**%**	**Ast**	**N**	**%**	
C	7	100	C	7	100	C	0	0	C	0	0	
F	0	0	F	0	0	F	7	100	F	2	100	
**Teeth**	**Incisors**		**Upper molars**		**Lower molars**							
	**N**	**%**	**N**	**%**	**N**	**%**						
C		40.6	112	80.6	70	84.3						
F	19	59.4	27	19.4	13	15.7						

Even though the bones were fractured while fresh, most long bone fractures were chemically altered by digestive gastric acids, and the typical features of green fractures (V-shaped and helical) were practically missing.

### Bone surface modifications

#### Digestive damage

In the sample 98.1% of the remains presented digestive damage, with 36.9% exhibiting ‘extreme’ digestion, 41.3% ‘heavy’ digestion and 16.8% ‘moderate’ digestive damage; light digestive damage was recorded only rarely (3.1%, Fig. 1 and Table 1). Different bones were altered in similar proportions, although patellae (because of their robustness) and some metapodials and phalanges (because they were still protected by part of the rabbit skin) displayed a lower degree of damage. Other bones, such as scapula, pelvis and astragalus fragments, were damaged to a slightly greater extent. Normally, the entire surface of the bones was affected by digestive corrosion (Fig. 1) as a result of the small size of the fragments. In addition, 100% of dental remains were altered. However, a bias towards slighter degrees of digestive damage in comparison to bone remains was observed (Table 1). No clear differences between incisors and upper and lower molars were in evidence.

#### Tooth marks

As a consequence of the high degree of digestive damage, tooth marks were practically inexistent. Only one tooth puncture (0.1% of the sample) was registered on the surface of a femur shaft fragment.

### Density-mediated attrition

A statistically significant correlation was found between bone mineral density^40^ and the frequency of the rabbit skeletal parts recovered in the sample of rabbit remains (rho = 0.6, p = 0.014). This indicates that the preservation of rabbit remains accumulated by the wolf may be affected by structural density-mediated attrition.

## Discussion

Distinguishing human and predator activities in faunal archaeological assemblages is a fundamental issue for understanding human subsistence in the past as well as site formation processes. In this framework, the data provided in this study are essential to recognizing the activities of the wolf in archaeological rabbit accumulations. The role of wolves as agents responsible for archaeofaunal assemblages has generated great interest among researchers^41–47^. In fact, the large number of taphonomic studies dedicated to wolves, along with the results obtained by studies about wolves’ behaviour demonstrating their capacity to accumulate prey remains through carcass transport and scats^37–39^, are proof of the important role that this carnivore may have played on archaeological assemblages. This is also confirmed by the archaeological evidence, a good example is the case of Denisova Cave where wolf fossil scats were recovered from different levels and chambers^48^. However, the interest in wolves has always focused on large prey such as cows, bison, horses, sheep, red deer or roe deer^41–47^. As a result, the taphonomic signal of wolves on small mammals such as rabbits has never been analysed until now.

The results reported in this study indicate that, unlike other smaller carnivores, such as coyotes, badgers, lynxes or foxes, the wolf ingests the entire prey carcass during feeding, with no portions of the rabbit resting unconsumed. Such behaviour will have generated assemblages comprising only ingested bones which, through the carnivore scats, may have accumulated in the same caves occupied by humans in the past. The lack of non-ingested leftovers in the small-prey assemblages generated by wolves differentiates them from all predators studied. All other predators, terrestrial carnivores and raptors alike, normally accumulate assemblages of mixed origin^19^, with different proportions of non-ingested and ingested remains from the predator’s scats or pellets. The greater or lesser presence of each type of remains determines the taphonomic pattern of predators. In the case of the wolf, because all the remains are from scats, their signature on rabbit bones will be more stable and easier to discriminate.

In accordance with the data recorded in the present study, rabbit assemblages accumulated by wolves will normally be described by the appearance of all skeletal elements, but with a significant prevalence of cranial elements – mostly teeth – and third phalanges, and a scarcity of carpal/tarsal bones, ribs and metapodials. The better representation of hind limbs than forelimbs is observed along with the high occurrence of fragmented and heavily digested bones, and a scarcity of tooth pits/punctures in the bones. Analysed in depth, the results of this research demonstrate that the taphonomic pattern left by wolves on rabbits differs from that of other predators.

To facilitate comparisons, Tables 3 and 4 present a summary of the results obtained from different rabbit predators (terrestrial carnivores and raptors), where the data have been collected using the same or similar methods.

**Table 3:** Anatomical representation, breakage, digestion and tooth marks for rabbit remains assemblages originated by different types of terrestrial carnivores compared with the results obtained for wolves in the present study. 19: Mallye *et al*. 2008; 21: Alvarez *et al*. 2012; 22: Lloveras *et al*. 2008; 24: Lloveras *et al*. 2012; 26: Rodríguez-Hidalgo *et al*. 2013; 27: Armstrong 2016; 49: Lloveras *et al*. 2018.

Terrestrial carnivores	Wolf *Canis lupus*	Coyote *Canis latrans*		Geoffroy’s cat *Leopardus geoffroyi*	Badger *Meles meles*	Fox*Vulpes vulpes*		Iberian lynx *Lynx pardinus*		European wildcat *Felis silvestris*	
References	Present study	27	21	19	24	22		26		49	
**Origin**	Scats	Scats	Non-ingested	Non-ingested	Mixed	Scats	Non-ingested	Scats	Non-ingested	Scats	Non-ingested
**N**	935	3903	771	793	812	265	639	1522	8772	87	1457
**MNI**	12	10	10	10	18	5	11	14	107	2	9
**RA% > values**	mol-cra-ph3	cra-lb	rib-ver	man-cra.inn	—	long bone-sc	mts-ast-tib	man-teeth-cra	tib-cal-mts	sc-hu-ra-ul-cr	cr-fe-mts-cal
**RA% < values**	c/t-rib	ver-rib	cra-phal	ver-mtc	—	mtc-c/t-inn	cr-sc-rib	c/t-ver-rib	sc-ver-hum	teeth-hindlimb	sc-rib-hu-ver
**Cranial/Postcranial**	+cranial	=	+postcranial	+cranial	+cranial	=	+postcranial	+cranial	+postcranial	+postcranial	+cranial
**Proximal/Distal**	+distal	+proximal	+proximal	=	—	+proximal	+distal	+proximal	+distal	+proximal	+distal
**Anterior/Posterior**	+hindlimb	=	+hindlimb	+hindlimb	+hindlimb	+hindlimb	+hindlimb	+forelimb	+hindlimb	+forelimb	+hindlimb
**PCRT/CR**	22.7 ± 0.4	50.6 ± 0.4	60.7 ± 0.5	40.5 ± 0.6	34.4 ± 3.7	55.5 ± 1.1	92.2 ± 0.4	27.9 ± 2.6	53.9 ± 0.2	60.5 ± 1.9	43.2 ± 0.4
**PCAP/CR**	24.6 ± 0.4	44 ± 0.3	30.1 ± 0.6	39.2 ± 0.6	37.7 ± 3.8	44.6 ± 1.3	91.8 ± 0.3	32.9 ± 2.8	49.6 ± 0.1	83.5 ± 1.8	38 ± 0.4
**PCLB/CR**	18.3 ± 1.3	56.8 ± 1.4	32.6 + ± 2.6	61.3 + ± 2	70.2 ± 3.6	77.8 ± 7.8	90.3 + ± 2	41.2 ± 2.9	62.7 ± 0.5	90.6 ± 4.8	44.2 ± 1.4
**AUT/ZE**	57.1 ± 1.3	46.8 ± 0.7	56.3 ± 1.7	27.2 ± 1.1	16.0 ± 2.9	19.7 ± 1.4	59.5 ± 1.2	40.3 ± 2.9	50.9 ± 0.3	30 ± 3.1	56.6 ± 0.8
**Z/E**	37.5 ± 9.6	51 ± 5	43.8 ± 12.3	47.7 ± 6.8	43.0 ± 3.9	37.9 ± 8.8	99.1 ± 3.8	44.6 ± 2.9	73.7 ± 1.7	57.1 ± 19.9	60.5 ± 5.7
**AN/PO**	37.5 ± 4.8	48.9 ± 1.7	56.5 ± 4.5	23.5 ± 2.2	32.5 ± 3.7	30.3 ± 4.3	22.6 ± 1.8	56.3 ± 2.9	38.9 ± 0.5	100 ± 0	42.4 ± 1.8
**Complete elements %**											
Mean value long bones	0	0	0	45.5	—	0	5.4	2.5	37.6	0	23.7
Mean value total	44.8	25.1	87.3	88.8	+50	12	89.4	43	73–78	11.5	92.3
**Length (in mm)**											
x̄ %<10 mm	7.4	7.9	15.6	—		9.3	19.3	7.1	17.4	5.4	21.5
%<10 mm	84.7	66.2	35.7	—		61	28	80	19.7	98.8	35
**% Digested remains**	98.1	94.4	0	0	14.3	99.5	0	97.2	0	98.6	0
**% Digested long bones**	100	—	—	—	—	100	—	100	—	100	—
**Degree**											
Null	1.9	6	—	—	—	0	—	2.8	—	1.4	—
Light	3.1	15	—	—	—	6	—	12	—	1.4	—
Moderate	16.8	30	—	—	—	26	—	22	—	9.6	—
Heavy	41.3	34	—	—	—	43	—	43.8	—	39.7	—
Extreme	36.9	14	—	—	—	25	—	19.3	—	47.9	—
**Teeth/beak pits & punctures**	0.1	0	2.3	19.8	4.7	3	9.5	0.3	0.9	0	1.2
**Age - % of adults**	—	—	—	—	80	87	—	21.4	—	—	—

**Table 4:** Anatomical representation, breakage, digestion and beak marks for rabbit remains assemblages originated by different types of raptors compared with the results obtained for wolves in the present study. 23: Lloveras *et al*. 2008; 27: Armstrong 2016; 50: Lloveras *et al*. 2014; 51: Lloveras *et al*. 2018; 58: Lloveras *et al*. 2009.

Raptors	Eagle owl *Bubo bubo*	Bald eagle *Haliaeetus leucocephalus*	S. Imperial eagle *Aquila adalberti*		Bonelli’s eagle *Aquila fasciata*		Great horned owl *Bubo virginianus*		Golden eagle *Aquila chrysaetos*	
Reference	58	27	23		50		27		51	
**Origin**	Nest	Non-ingested	Pellets	Pellets	Nest	Pellets	Non-ingested	Pellets	Nest	Pellet
**N**	1808	249	2275	824	438	193	264	3184	1543	670
**NMI**	19	—	10	16	9	4	—	10	16	9
**RA% > values**	cal-inn-fem	Rib-ver-pat	lb-rib	phal 3-u mol-tib	cra-u mol-inn	mol-cra-in	rib-pat-ver	ver-rib-cra	inn-cal-tib	cal-phal3
**RA% < values**	mtc-c/t	—	ver-phal	rib-fem-rad	mtc-rib	Pat-ast	—	mt-phal	rib-c/t	rib-c/t
**Cranial/Postcranial**	+postcranial	—	+postcranial	+cranial	+cranial	+cranial	—	+cranial	+cranial	+cranial
**Proximal/Distal**	+proximal	=	+proximal	+distal	+proximal	—	—	=	+proximal	+proximal
**Anterior/Posterior**	+hindlimb	+forelimb	+forelimb	+hindlimb	+hindlimb	—	+forelimb	+forelimb	+hindlimb	+hindlimb
**PCRT/CR**	60.6 ± 2.7	—	50.7 ± 0.3	27.7 ± 3.2	24.3 ± 0.5	24.1 ± 0.7	—	53.3 ± 0.3	34.7 ± 0.3	41.1 ± 0.6
**PCAP/CR**	64.7 ± 2.6	—	40.7 ± 0.4	36.3 ± 3.5	17.5 ± 0.5	21.1 ± 0.7	—	42.7 ± 0.4	27.4 ± 0.3	35.6 ± 0.6
**PCLB/CR**	70.0 ± 2.5	—	50.1 + ± 1.3	32.7 ± 3.4	32.5 ± 2	30.6 ± 3.1	—	52.7 + ± 1.2	43.7 ± 1.2	48.7 ± 2.2
**AUT/ZE**	38.8 ± 2.7	—	50.8 ± 0.7	55.4 ± 3.6	36.6 ± 1.6	50.6 ± 2.5	—	49.7 ± 0.7	42.4 ± 0.8	45.7 ± 1.2
**Z/E**	40.0 ± 2.7	—	49.6 ± 4.6	75.3 ± 3.1	44.4 ± 9.5	52.6 ± 16.8	—	49.6 ± 4.6	50 ± 5	43.2 ± 7.9
**AN/PO**	21.7 ± 2.3	—	49 ± 1.6	28.1 ± 3.2	30.2 ± 3.7	40.3 ± 5.9	—	50.3 ± 1.6	27.6 ± 1.4	31.3 ± 2.7
**Complete elements %**										
Mean value long bones	14.6	92.3	0	0	51.7	15	98.7	0	45.3	0
Mean value total	53.9	76.8	47.1	27	74.7	59.6	85.6	24.1	68.2	39.8
**Length (in mm)**										
x̄	14.5	29.7	7	8.4	19.7	8.3	31.7	9.1	23.4	10
%<10 mm	49	24.6	77.8	73	54.9	78.1	32.8	55.6	44.7	78.6
**% Digested remains**	68.8	0	96	98	31.2	72	0	72.5	32	73.6
**% Digested long bones**	88.9	—	—	100	31	—	—	—	50.3	—
**Degree**										
Null	31.2	—	4	2	68.8	27.9	—	27	68	26.4
Light	40.2	—	18	18.2	2.3	5.4	—	42	1.4	3.1
Moderate	19.8	—	32	46.8	7.9	18.3	—	22	4.3	9.9
Heavy	8	—	34	27.4	14.4	33.3	—	8	8.1	18.7
Extreme	0.7	—	12	5.6	6.5	15.1	—	1	18.2	41.9
**Teeth/beak pits & punctures**	2	0.6	0	0.5	0.8	0	2.3	0	1.1	0.1
**Age - % of adults**	50	—	—	—	41.4	—	—	—	83.5	—

Regarding anatomical representation, the profiles of the relative abundance of skeletal elements obtained for the wolf differ clearly from those of non-ingested and nest assemblages created by both terrestrial carnivores and raptors (Fig. 2). In the wolf sample, most skeletal elements display lower relative abundance values, which is to be expected given that non-ingested remains are always better preserved and consequently better represented. Compared to all other terrestrial carnivores apart from the wildcat, the only exception is the percentage of teeth, which is markedly higher in the wolf assemblage. The wildcat creates rabbit assemblages practically opposite to those of the wolf, with very large quantities of non-ingested remains^49^. Profiles of relative abundance for the wolf, wildcat, lynx and fox show that wildcats consume little of the rabbit skeleton, whereas the Iberian lynx is situated in an intermediate position followed by the red fox. The wolf is the carnivore that produces the greatest bone destruction, ingesting the whole carcass.

Compared to the nest samples of raptors, teeth are again the only skeletal element that is equally or better represented in wolves. The differences are most evident in the low values for long bones (except humeri), scapulae and innominates in the wolf accumulation (Fig. 2).

The anatomical representation profile for the wolf is closer to that of other predators when pellet or scat assemblages are considered; however, the wolf values continue to be different. In the case of remains from scats, the wolf displays higher values for molars and third phalanges, but other skeletal elements tend to be less represented, by contrast with the wildcat sample, which differs because in this case many rabbit bones are not ingested and therefore not represented^49^. Comparison with raptor pellet samples also shows differences. In the wolf accumulation, cranial fragments and teeth are more abundant whereas the long bones are normally scarcer.

These differences in the survivorship of skeletal elements in wolves compared to scat and pellet samples generated by other taxa were assessed using the chi-square test of independence, showing that the differences are statistically significant in all the cases tested (the p-value is always <0.00001, the result is significant at p < 0.05, Table 5). In general, the disparities registered in the anatomical representation profiles of the various predators examined are a consequence of the different feeding behaviours of predators.

**Table 5:** Above: chi-square and p-values obtained in the chi-square tests of independence applied to assess differences in the survivorship of skeletal elements or their fragments in wolves compared to scat and pellet samples generated by other taxa. Below: factorial matrix for components 1 and 2.

	Chi-squared test		
	χ^2^	p-value	df
wolf-fox	15307.8	< 0.00001	16
wolf-lynx	20476.4	< 0.00001	16
wolf-wildcat	13330.1	< 0.00001	16
wolf-coyote	26227.8	< 0.00001	16
wolf-imperial eagle	3182.9	< 0.00001	16
wolf-Bonelli’s eagle	3072.1	< 0.00001	16
wolf-golden eagle	3800.8	< 0.00001	16
	**PCA - Factorial matrix**		
	**Component 1**	**Component 2**	
complete long bones	−0.667	0.74	
complete remains	−0.918	−0.103	
average length	−0.791	−0.062	
remains <10 mm	0.907	0.223	
digested remains	0.989	0.103	
digestion 1	0.439	−0.132	
digestion 2	0.814	0.009	
digestion 3	0.905	0.173	
digestion 4	0.689	0.198	
pits & punctures	−0.443	−0.471	

With regards to bone fragmentation, analysis of breakage patterns reveals a high amount of destruction in the wolf assemblage: 84.7% of remains measured less than 10 mm and the percentage of complete bones was 44.8%, whereas the long bones were always fragmented. In the case of terrestrial carnivores such as the wildcat, Iberian lynx, coyote, Geoffroy’s cat and the red fox, rabbit assemblages of non-ingested remains are defined by a low degree of breakage (Table 3). The percentage of remains under 10 mm does not exceed 35% and the percentage of complete elements is always close to 80% or higher. This situation changes completely in the scat assemblages of terrestrial carnivores. Rabbit fragments from wolf scats are very close in size to those from Iberian lynx samples, a little smaller than in the scats of coyote and fox, and slightly larger than those from wildcat scats. Similarly, the percentage of complete elements is analogous in the lynx sample but lower in the rest of the carnivores (Table 3). The similarities in the degree of breakage in wolf and Iberian lynx scat samples are related to the high numbers of small-sized elements, mostly teeth and third phalanges, recovered in these assemblages. Because of their small size, these elements are often recovered complete, increasing the percentage values of complete elements. However, in all scat accumulations, breakage patterns are similar, and considering that the completeness values may vary slightly as a consequence of intraspecific variables (the age of the prey, age of the predator, rabbit abundance, etc.)^25,26^ the values obtained for different carnivores could overlap, making distinctions difficult.

Similarly to what occurs with terrestrial carnivores, the high degree of breakage observed in the wolf sample diverges from the values obtained in the nest assemblages of raptors, which contain large quantities of complete non-ingested remains (Table 4). However, the breakage patterns observed in pellet accumulations are again very similar to that of the wolf. The main difference is in the percentage of remains under 10 mm, which in pellet samples never reaches 80% (the highest value is 78.6% for the golden eagle), whereas in the wolf the figure is almost 85%. Another difference, related to long bone fragmentation, is the high representation in the wolf assemblage of fragments containing the proximal epiphysis, which clearly outnumber the distal ends (Table 2). In raptors such as imperial eagles, Bonelli’s eagles or golden eagles, both categories, proximal and distal fragments alike, are more or less equally present^23,50,51^, and in the scats of Iberian lynxes and foxes, proximal and distal epiphysis fragments are also present in equal measure by contrast with the wolf results^22,24^.

When feeding on rabbit carcasses, the different types of predators produce much higher percentages of tooth/beak damage in nest samples and samples of non-ingested remains (Tables 3 and 4). In scat and pellet accumulations, the high degree of bone destruction affects tooth/beak marks. As mentioned above, these samples are highly fragmented, and marks become distorted or eliminated when bones pass through the digestive system of the predator; for this reason pits and punctures are rare. The present study has shown this to be the case with rabbit assemblages generated by wolves, for we found only 0.1% of bones to be tooth marked, which is similar among all samples of ingested remains. The only exception is the red fox, which produces higher numbers of marks (2%) than the rest of the predators studied (Table 3).

Regarding digestive damage, the proportion of digested elements in the wolf sample (98.1%) is higher than that obtained for raptor nest assemblages (i.e. 32% for the golden eagle; 31.2% for Bonelli’s eagle, Table 4), but very close to the number of digested remains in some raptor pellets (i.e. 98% for the imperial eagle) and all carnivore scat assemblages, in which almost 100% of the remains display digestive corrosion. In the wolf sample, however, digestive damage is more pronounced than in the raptor samples, with a higher percentage of remains damaged to a heavy or extreme degree (78% in total). Damage caused by digestion is also more marked in the wolf sample than in the coyote, red fox and Iberian lynx scat accumulations, but slightly lower than in the wildcat assemblage (78% vs 87%, Table 3).

The results provided in this study make evident the differences between wolf and anthropogenic contributions. Differences are multiple, among them, the lack in human originated assemblages of digested remains or the presence of significant proportions of long bone cylinders associated to a pattern of bone marrow consumption, cut marks and thermo-altered bones, are some of the most remarkable^52^.

Finally, rabbits are a gregarious species that construct warrens where they live in large groups. For this reason, when analysing archaeological rabbit remains it should be taken into account they could be intrusive, as a result of natural death in their burrows. According to Pelletier *et al*.^53^, warren rabbit accumulations are characterized by: a large number of infant individuals, a major presence of forelimb bones, a moderate breakage with a high proportion of dry breaks, and no traces of predation (digestion, tooth or cut marks, etc.). Our results clearly differ, the high degree of breakage and digested remains registered move away without any doubt from the evidence of warren samples.

**Figure 3:**
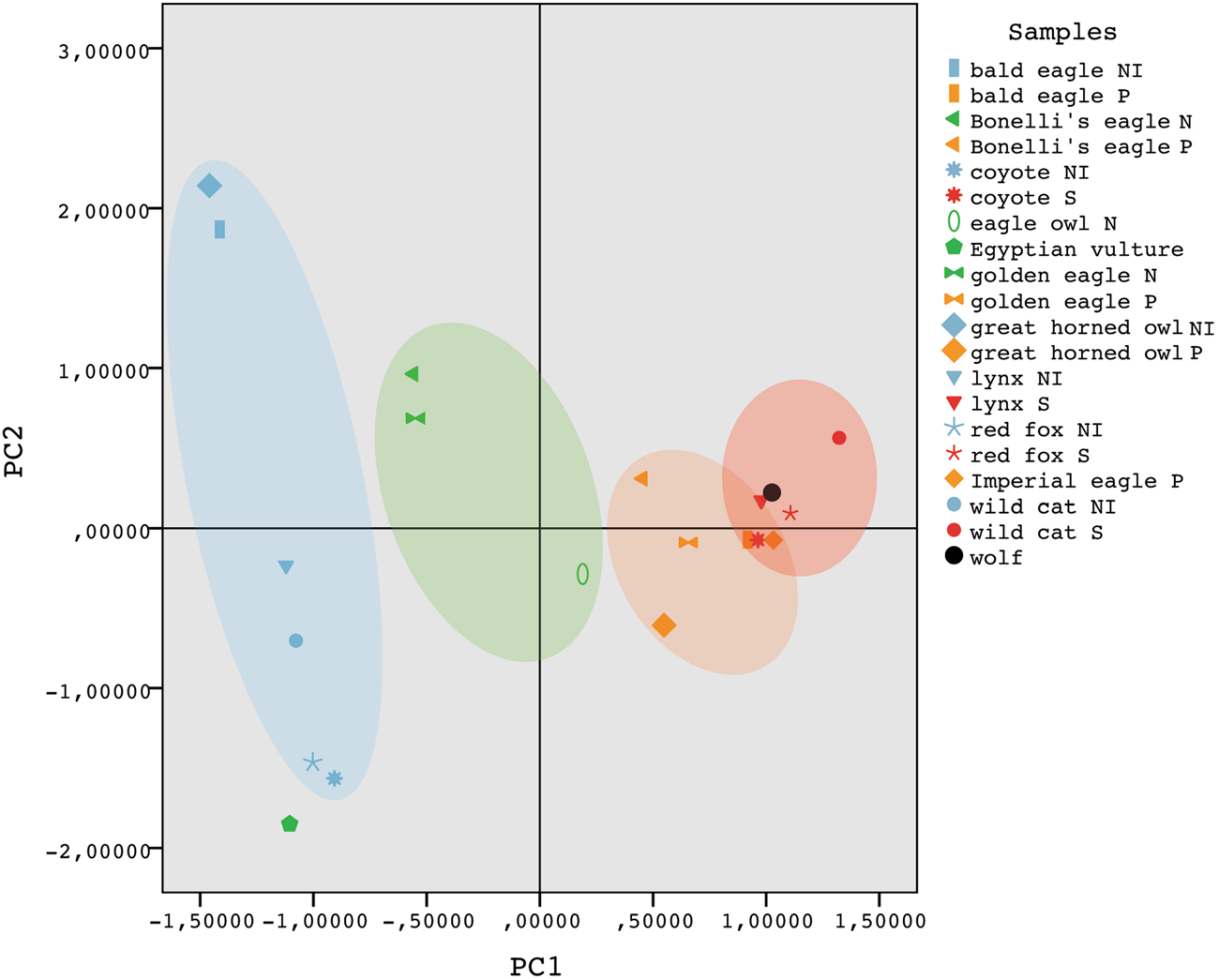
Scatter plot of the principal component analysis results for different types of assemblages of rabbit remains accumulated by terrestrial carnivores and raptors. The elliptical coloured areas group the samples according to their origin: non-ingested (NI), nest (N), pellet (P) and scat (S). Variance observed: 76.56% PC1 and 11.53% PC2.

In order to go deeper in our investigation of the taphonomic signature of wolves on rabbit assemblages, we used the principal component analysis (PCA) to examine this sample together with a set of different samples of ingested and non-ingested rabbit remains. PCA is a multivariate statistical method that simplifies the complexity in high-dimensional data while retaining trends and patterns. It does this by transforming the data into fewer dimensions, the principal components, which act as summaries of features^54^. The variables considered in the analysis were: the percentage of complete bones and complete long bones, the average length of rabbit remains, the percentage of elements under 10 mm, the presence and degree of digestion, and tooth/beak marks. The PCA results reduced the variables analysed to two factors (PC1 and PC2, see Table 5), the PC1 explain 76.56% of the variance observed and the PC2 11.53%. The first component accounts mostly for complete elements, remains < 10 mm, and digestion. The second component accounts mostly for tooth/beak marks and complete long bones. The distribution of the samples analysed is represented in the scatterplot of Fig. 3. PC1 differentiates between ingested and non-ingested accumulations, situating the non-ingested assemblages in the negative region (lower scores) in opposition to the scats and pellets, which are located in the positive region. Consequently, assemblages of mixed origin, such as nest samples, are positioned in the middle region. The only sample that is classified outside the area that would correspond to it is the Egyptian vulture nest assemblage. This is probably because this bird is a scavenger, and its feeding behaviour is very different from all other raptors analysed^55,56^. The scatterplot shows that there is some overlap among pellet and scat accumulations. However, both types of sample may normally be differentiated because terrestrial carnivores tend to display higher values for the presence and degree of digestion damage and for small-sized elements. Within each group, moreover, PC2 situates assemblages with a larger number of complete long bones above, in the positive region, whereas those samples with a greater number of tooth/beak punctures are placed below, in the negative region.

The wolf sample is situated among the other scat assemblages, very close to the Iberian lynx and the rest of the terrestrial carnivores. Despite these similarities, the principal component analysis shows that there are some tangible differences among the samples, further demonstrating that the predators handle small-prey carcasses distinctively.

Our detailed taphonomic analysis of the rabbit remains accumulated by wolves demonstrates that it is possible to distinguish samples generated by this predator from those created by humans and by other carnivores. First of all, the rabbit assemblages created by wolves can be differentiated in that they are only composed of ingested remains, whereas all other predators normally accumulate samples including a mixture of ingested and non-ingested skeletal elements. Moreover, the combined suite of anatomical representation profiles, degrees of breakage and frequencies of bone surface modification provided here also makes it possible to discriminate wolf samples from similar scat or pellet accumulations created by other predators.

Some variables, such as the age of the prey, may introduce variability in the results obtained in actualistic studies. The rabbits used in this work were subadults, which together with adults, use to be the preferred age prey of most predators. However, the preferred age is also a feature that varies depending on different factors such as prey availability or season of capture. Some studies have demonstrated that variability related to the age of the rabbits is less than originally thought^25^, indicating the validity of our results to detect the activity of wolves in rabbit archaeological assemblages. However, researchers have also warned that studies conducted with captive carnivores may be biased because the animal behaviour and their resulting bone modification patterns may vary^57^. For this reason, the biases that this and other variables (e.g. age, sex and number of predators) could introduce to predator taphonomic signatures need to be further investigated.

## Methods

The wolf scats used in the study were rehydrated, water-screened and disaggregated in a 1.5 mm mesh in order to recover all the skeletal elements that they contained. The analytical methodology used in this study follows the criteria applied in previous works carried out with leporid assemblages generated by different predators^22–24,50,51,58^. The variables considered within each of the analytical parameters studied are presented below.

### Anatomical representation

The number of identified specimens (NISP), the minimum number of elements (MNE) and the minimum number of individuals (MNI) were calculated, as well as relative frequencies. Relative abundance (RA) was calculated using the formula advocated by Dodson and Wexlar^59^. In addition, proportions of skeletal elements were evaluated using the following ratios^16,22,53^:

- PCRT/CR = [(PCRT × 32)/((PCRT × 32) + (CR × 184))] × 100 with PCRT being the total number of postcranial elements (limbs, vertebrae and ribs) and CR the total number of cranial elements (mandibles, maxillae and teeth);
- PCRAP/CR = [(PCRAP × 32)/((PCRAP × 32) + (CR × 114))] × 100 with PCRAP being the total number of limb elements (long bones, scapulae, innominate, patellae, metapodials, carpals, tarsals and phalanges);
- PCRLB/CR = [(PCRLB × 32)/((PCRLB × 32)+(CR × 10))] × 100 with PCRLB calculated as the total number of long bones (humerus, radius, ulna, femur and tibia);
- AUT/ZE = [(AUT × 12)/((AUT × 12) + (ZE × 98))] × 100 with AUT being autopodia (metapodials, carpals, tarsals and phalanges) and ZE zygopodia and stylopodia (tibiae, radii, ulnae, humeri, femora and patellae);
- Z/E = [(Z × 4)/((Z × 4) + (E × 6))] × 100 with Z referring to zygopodia (tibiae, radii and ulnae) and E stylopodia (femora and humeri);
- AN/PO = [(AN × 12)/((AN × 12) + (PO × 16))] × 100 with AN representing the number of scapulae, humeri, radii, ulnae and metacarpals and PO innominates, femora, tibiae and metatarsals.
- The 95% confidence intervals have been calculated for each proportion index.

### Breakage

The breakage pattern was described in terms of the maximum length of all identified skeletal elements. Percentages of complete elements, isolated teeth and articulated elements were calculated. For immature individuals, the diaphyses of long bones with unfused epiphyses were considered complete elements. Bone fragments were categorised according to bone type:

- Patellae, carpals, tarsals and ribs were classified as complete (C) or fragmented (F).
- Phalanges were recorded as complete (C), or proximal (P) or distal (D) fragments. When the distinction between proximal or distal was not possible, they were recorded as fragments (F).
- Vertebrae were registered as complete (C), vertebral body (VB), vertebral epiphysis (VE) or spinous process (SP).
- Breakage of teeth was calculated separately for isolated and *in situ* elements^60^, and the teeth were classified as complete (C) or fragmented (F).

Breakage categories for long bones, metapodials, mandibles, crania, scapulae and innominates are fully described and illustrated in Lloveras *et al*. (Fig. 1)^22^. The presence of long bone cylinders (fragments of long bones with snapped ends resulting from consumption) and V-shaped and helical fractures^27,61^ were also recorded.

### Bone surface modifications

All of the skeletal remains were examined both macro- and microscopically. Damage to the bone surface was observed under a light microscope (x10–x40 magnification) with an oblique cold-light source.

#### Digestive damage

Different categories of digestive damage were applied to bones and teeth^22,23,60^. Five categories of digestion were distinguished: null (0); light (1); moderate (2); heavy (3); and extreme (4). These were valued separately for bones and dental remains.

#### Tooth marks

Damage to bone surfaces caused by teeth was noted and counted. Marks were classified as scoring, notches, tooth punctures/tooth pits and crenulated/fractured edges^36,41^. Punctures and pits were also classified by their number (isolated or multiple) and distribution (unilateral – i.e. located on one surface – or bilateral)^56^.

### Density-mediated attrition

Differential survival in relation to bone density was evaluated using the bivariate *Spearman’s rho* correlation^62^, taking into account the data on rabbit bone density provided by Pavao and Stahl^40^.

## Data Availability

The datasets supporting the findings of this study are available within the article and from the corresponding author on request.
